# Determinants and effects of positive surgical margins after prostatectomy on prostate cancer mortality: a population-based study

**DOI:** 10.1186/1471-2490-14-86

**Published:** 2014-11-05

**Authors:** Valesca P Retèl, Christine Bouchardy, Massimo Usel, Isabelle Neyroud-Caspar, Franz Schmidlin, Gregory Wirth, Christophe Iselin, Raymond Miralbell, Elisabetta Rapiti

**Affiliations:** Geneva Cancer Registry, Global Health Institute, University of Geneva, 55 Bd. de la Cluse, Geneva, 1205 Switzerland; Urological Centre, Clinique des Grangettes, chemin des Grangettes 7, Chêne-Bougeries, 1224 Switzerland; Department of Urology, Geneva University Hospitals, rue Gabrielle Perret-Gentil 4, Geneva, 1205 Switzerland; Department of Radiation Oncology, Geneva University Hospitals, rue Willy Donzé 6, Geneva, 1205 Switzerland

**Keywords:** Prostate cancer, Prostate cancer-specific survival, Prostatectomy, Surgical margins

## Abstract

**Background:**

The objective of this population-based study was to assess patient, physician and tumour determinants associated with positive surgical margins after prostatectomy, and to assess the effects of positive surgical margins on prostate cancer-specific survival.

**Methods:**

We included 1’254 prostate cancer patients recorded at the Geneva Cancer Registry who had radical prostatectomy during 1990–2008. To assess factors associated with positive margins, we used logistic regression. We assessed the effects of positive margins on prostate cancer-specific survival by Cox proportional hazard models accounting for numerous other prognostics factors including prostate and tumour volume, the total percentage of tumour, radiotherapy, surgical approach and surgeon’s caseload.

**Results:**

Among men undergoing prostatectomy, 479 (38%) had positive margins. In the multivariate logistic regression analysis, period, clinical- and pathological T stage, Prostate Specific Antigen (PSA) level, Gleason score and percentage of tumour in the prostate were significantly associated to positive margins. Ten-year prostate cancer-specific survival was 96.6% for the negative margins group and 92.0% for the positive margins group (log rank p = 0.008). In the Cox survival analysis adjusted for tumour characteristics, surgical margin status *per se* was not an independent prognostic factor while age, pathological T, PSA level and Gleason score remained associated with prostate cancer-specific survival.

**Conclusions:**

More aggressive tumour characteristics were strong determinants for positive margins. Furthermore, surgical margin status *per se* was not an independent prognostic factor for prostate cancer-specific survival after adjusting by the gravity of the disease in the multivariate analysis.

## Background

Prostate cancer is the second most frequently diagnosed cancer and the fifth most common fatal cancer among men worldwide, the fourth for developed countries [1]. A high percentage of men are diagnosed with early stage disease due to the spontaneous generalization of prostate specific antigen (PSA) screening [2]. Radical prostatectomy is the main treatment option for clinically localized prostate cancer, as shown by the randomized Scandinavian prostate Cancer Group Study Number 4 (SPCG-4) [3]. However, as shown recently by Wilt *et al*., the difference between radical prostatectomy and watchful waiting was not significant [4]. Positive surgical margins (PSMs), identified as the presence of cancer at the inked resection margin of the prostatectomy specimen, are a relatively frequent finding after prostatectomy and reported in many articles with proportions ranging from 10 to 40% [5]. Although there are many reports and studies available, questions remain regarding determinants and effects of PSM. Furthermore, in relation to survival, surgical margins are the only factor which could be influenced by the surgeon, contrary to tumour characteristics.

Some investigations have correlated patient, clinical and histo-pathological findings with surgical margin status after prostatectomy [6–8]. Coelho *et al*., investigated several determinants of surgical margins, such as type of surgery, location of PSM, prostate weight and tumour volume [6]; Williams *et al*., investigated determinants such as period of surgery, geographical region, type of surgery and surgeon caseload [7]; and finally Vesey *et al*., studied surgeon caseload and prostate weight [8]. They all found that the tumour characteristics such as clinical stage, pathological stage, Gleason score and the percentage of tumour in the prostate were more often significantly associated with surgical margin status.

Furthermore, several studies have investigated the association between surgical margins and outcome after radical prostatectomy [9–17]. Two types of outcomes are usually described in this context; either PSA biochemical recurrence (BCR) through routine serum PSA monitoring every six months after treatment, and/or prostate cancer-specific survival (PCSS). PSM after radical prostatectomy is known to be an independent risk factor for BCR; however, there is no wide consensus on its impact on survival [14–16]. Although most studies reporting on BCR show significant association with PSM [9–12], studies reporting on PCSS show mostly non-significant association with PSM [13–17].

The aim of the present study was twofold; 1) to assess patient, physician and tumour characteristics associated with PSM after prostatectomy, and 2) to assess if PSM is an independent prognostic factor for prostate cancer-specific mortality (PCSS).

## Methods

### Patients

We included 1’254 patients diagnosed with prostate cancer between 1990 and 2008 who underwent radical prostatectomy using data from the Geneva Cancer Registry in Switzerland.

### Variables

The Geneva Cancer Registry records all incident cancers occurring in the population of the canton (approximately 475’000 inhabitants in 2009). All hospitals, pathology laboratories, and practitioners are requested to report all cancer patients. Trained registrars systematically abstract data from medical and laboratory records. Physicians regularly receive questionnaires to obtain missing data. Recorded data include various information on patient and tumour characteristics, treatment and outcome. The Registry regularly assesses survival data. Active follow-up is performed yearly using the files of the Cantonal Population Office which is in charge of registration of the resident population. The registrars establish cause of death by systematically consulting clinical records and interpreting questionnaires completed by the patient’s physician. In general, the Registry records data on treatments given during the first six months, however for this study we re-opened all files and collected all treatment data. The patients were followed for vital status up to 31^rst^ December 2011.

As variables of interest, we considered age in continuous or categorical (<60, 60–69, ≥70 years), period at diagnosis (1990–4, 1995–9, 2000–4, 2005–8), socioeconomic status based on last patient’s occupation (high, middle, low, unknown), sector of care (private, public), method of detection (screening by PSA, symptoms, other), and surgeon caseload (the mean of prostate cancer surgeries that the surgeons performed). For surgical approach we considered "open" procedures (perineal and retropubic), laparoscopic and robot assisted laparoscopic surgeries. Because before 1998 laparoscopic and robot assisted prostatectomies were not performed in Geneva, we classified the "unknown" procedures before 1998 as "open" surgical procedures. Adjuvant radiotherapy was given in adjunction (within one year) to prostatectomy, without signs of PSA raise. Salvage radiotherapy was given only after PSA raise or other signs of local recurrence. In the analyses, the variable radiotherapy includes both those patients with adjuvant radiotherapy and those with salvage radiotherapy. In case radiotherapy was accompanied by hormonal therapy, this was included in the radiotherapy category.

All specimens were analysed by one of the three pathology laboratories existing in Geneva; one public and two private. The presence of tumour cells at the inked margin of the resection was considered to represent a PSM. A "close" surgical margin was considered as tumour cells in close proximity to the ink (within 1 mm from the inked margin), according to Chuang and Epstein [18]. Besides the surgical margin status, other tumour characteristics were considered such as clinical stage (cT0-cT1, cT2, cT3, cTx (unknown) and pathological stage (pT1-2, pT3-T4) [19], PSA value at diagnosis (<10 ng/ml, 10–20, >20), Gleason score (Gleason < 7, Gleason = 7, Gleason > 7), margin extension as focal (unifocal and < = 3 mm) and extensive (plurifocal and >4 mm), and tumour percentage in the prostate. The volume of the prostate and of the tumour were measured by multiplying the largest height, width and length by 0.524 (*H* × *W* × *L* × π/6), according to Bates *et al*. [20] Tumour percentage of the prostate was calculated as tumour volume divided by the prostate volume. As capsular extension was highly correlated with pathological T status (intracapsular = pT1-2, extracapsular extension = pT3-4), we did not include this variable in our analysis.

### Statistical analysis

We performed a case–control study, considering patients with PSM as cases and patients with negative surgical margins as controls. We used the *χ*^2^ test and univariate and multivariate logistic regression to assess the relationship between surgical margins and other variables. Ten-year prostate cancer survival curves according to surgical margins status were estimated by means of Kaplan Meyer methods, and survival differences were tested through log rank test. We used Cox proportional regression analyses to assess the independent effect of surgical margins on ten-year prostate cancer-specific survival after radical prostatectomy while adjusting for other prognostic factors as assessed in univariate analysis. In the logistic and Cox regressions we regrouped negative-and close surgical margins together for further analyses as suggested in the literature [21]. The statistical significance level was set at *p* value < 0.05. Stepwise backward elimination was used to reduce the model to factors with p < 0.10. Statistical analysis was performed using the software Statistical Package for Social Sciences (SPSS version 15.0, Chicago, IL).

All data analysis was conducted at the Geneva Cancer registry. As a cancer registry we do not require Ethical permission and accordingly no informed consent is required for registry-based studies involving no contact with the study subjects. The Registry has a general authorization, provided by the Federal Expert Commission in charge of data protection, to collect nominative data for research purposes on cancer in the general population.

## Results

### Association between patient and tumour characteristics and post-operative surgical margins

Among the 1’254 prostate cancer patients undergoing prostatectomy, 479 (38.2%) had PSM, 629 (50.2%) negative surgical margins and 146 (11.6%) close surgical margins. We observed that the most common location of PSM was at the apex (53.9%) and the posterior (19.0%) of the prostate (data not shown).

Table 1 presents the distribution of patient characteristics, tumour characteristics, and treatment according to surgical margins and the Odds Ratios (ORs) derived from the univariate logistic regression. The patients with negative and close margins were considered together in the category negative margins. The mean age at diagnosis was 63 years (Standard deviation [SD] ±6.3 years) and did not differ between the two groups of surgical margins. The PSM rate decreased over time, from 50% in 1990–1994 to 31% in 2005–2008 (test for trend p < 0.001). Most patients were treated in the private sector (64.1%). The majority of the patients were diagnosed through screening (83.4%). Close surgical margins were more often reported by the private pathology laboratories compared to the public ones (71.9% and 28.1% respectively, p = 0.049). The information about lymph node dissection was available only since 1999. Among the 1023 patients for whom we had the information, 374 (37%) had a lymph node dissection, and of these 347 were free of metastasis (214 in the negative and 133 in the positive margin group). In terms of PSM rates were higher after the open surgical procedures (196/427, 45.7%) compared to robot-assisted prostatectomies (35/109, 32.1%) (p = 0.010). Among the 479 patients with PSM, 32 (7%) received adjuvant radiotherapy, and 124 (26%) received salvage radiotherapy. For the patients with negative surgical margins, adjuvant radiotherapy was given to only 3 of 775 (0.4%) patients, and 30 (4%) received salvage therapy. In the univariate analysis, all tumour characteristics such as clinical and pathological T-status, PSA level, Gleason score, prostate volume, tumour volume and percentage were significantly associated with PSM.

**Table 1:** **Comparison** ^**a**^ **of sociodemographic, treatment and surgeon characteristics among prostate cancer patients after prostatectomy (n = 1’254) according to surgical margin status**

Characteristics	Surgical margin status				Total	Crude	P-value
						Odds ratio	
	Positive		Negative ^b^				
	(cases)		(controls)				
	N	%	N	%	N	(95% CI)	
**Age** Mean (SD)	63.39	(6.00)	62.94	(6.48)	63.12	(6.30)	0.218
							(*t*-test)
**Age class**							
<60	127	37.1	215	62.9	342	1	0.831
60-69	276	38.3	445	61.7	721	1.05 (0.81-1.37)	
≥70	76	39.8	115	60.2	191	1.12 (0.78-1.61)	
**Period**							
1990-1994	29	50.0	29	50.0	58	1	<0.001
1995-1999	105	42.9	140	57.1	245	0.75 (0.42-1.33)	
2000-2004	201	40.8	292	59.2	493	0.69 (0.40-1.19)	
2005-2008	144	31.4	314	68.6	458	0.46** (0.26-0.80)	
**Social class status**							
High	156	35.9	279	64.1	435	1	0.602
Middle	206	38.8	325	61.2	531	1.13 (0.87-1.47)	
Low	107	40.8	155	59.2	262	1.24 (0.90-1.69)	
Unknown	10	38.5	16	61.5	26	1.12 (0.50-2.52)	
**Sector of care**							
Private	312	38.8	492	61.2	804	1	0.554
Public	167	37.1	283	62.9	450	0.93 (0.73-1.18)	
**Method of detection**							
Screening	394	37.7	652	62.3	1046	1	0.434
Symptoms	43	44.3	54	55.7	97	1.32 (0.87-2.01)	
Other	42	37.8	69	62.2	111	1.01 (0.67-1.51)	
**Type of surgery**							
Perineal/Retropubic	196	45.9	231	54.1	427	1	<0.001
Laparoscopic	62	39.2	96	60.8	158	0.76 (0.53-1.10)	
Robot-assisted	35	32.1	74	67.9	109	0.56* (0.36-0.87)	
Unknown	186	33.2	374	66.8	560	0.59*** (0.45-0.76)	
**Surgeon caseload (number of prostatectomies)**							
High (>50)	379	37.6	628	62.4	1007	1	0.235
Middle (15–50)	74	44.6	92	55.4	166	1.33 (0.96-1.86)	
Low (<15)	24	32.0	51	68.0	75	0.78 (0.47-1.29)	
Unknown	2	33.3	4	66.7	6	0.83 (0.15-4.55)	
**Radiotherapy with or without hormonal therapy** ^**c**^							
No	325	30.5	742	69.5	1067	1	<0.001
Yes	154	82.4	33	17.6	187	10.65 (7.16-15.86)	
**Clinical T**							
cT0-cT1	83	31.8	178	68.2	261	1	<0.001
cT2	163	35.7	293	64.3	456	1.19 (0.86-1.65)	
cT3	79	63.2	46	36.8	125	3.68*** (2.36-5.76)	
cTx	154	37.4	258	62.6	412	1.28 (0.92-1.78)	
**Pathological T**							
pT0-pT2	239	27.5	629	72.5	868	1	<0.001
pT3-pT4	240	62.2	146	37.8	386	4.33 (3.36-5.58)	
**PSA level**							
0-9	217	32.8	445	67.2	662	1	<0.001
10-20	98	51.0	94	49.0	192	2.14*** (1.54-2.96)	
21+	57	64.0	32	36.0	89	3.65*** (2.30-5.80)	
Unknown	107	34.4	204	65.6	311	1.08 (0.81-1.43)	
**Gleason**							
2-6	218	30.5	497	69.5	715	1	<0.001
7	195	45.2	236	54.8	431	1.88*** (1.47-2.41)	
8-10	60	61.9	37	38.1	97	3.70*** (2.37-5.74)	
Unknown	6	54.5	5	45.5	11	2.74 (0.83-9.06)	
**Prostate volume (cm** ^**3**^ **)**							
≤60	422	41.0	607	59.0	1029	1	<0.001
>60	53	25.7	153	74.3	206	0.50*** (0.36-0.70)	
Unknown	4	21.1	15	78.9	19	0.38 (0.13-1.16)	
**Tumour volume (cm** ^**3**^ **)**							
≤10	347	35.7	624	64.3	971	1	<0.001
>10	58	70.7	24	29.3	82	4.35*** (2.65-7.12)	
Unknown	74	36.8	127	63.2	201	1.05 (0.76-1.44)	
**Tumour percentage** ^**d**^							
≤10%	202	26.7	554	73.3	756	1	<0.001
>10%	252	57.9	183	42.1	435	3.78*** (2.94-4.85)	
Unknown	25	39.7	38	60.3	63	1.80* (1.06-3.07)	
**Total**	**479**	**38.2**	**775**	**61.8**	**1245**		

Geneva Cancer Registry 1990–2008.

^a^Crude logistic regression comparing patients with positive surgical margins (the cases) with those with negative surgical margins (the controls).

^b^Negative and close.

^c^Radiotherapy includes both salvage and adjuvant categories.

^d^Tumour percentage of the prostate was calculated as tumour volume divided by the prostate volume

CI: confidence interval.

*p < 0.05; **p < 0.01; ***p < 0.001.

Table 2 presents the results from the multi-adjusted logistic regression model. All factors significantly associated with PSM in the univariate analysis were simultaneously entered in the model. Clinical and pathological T status were entered into two separate models, due to their high co-linearity, the results presented derive from the model with clinical T status. The following factors remained significantly associated with PSM: period of diagnosis, clinical T3 status compared to the cT0-1 reference category (Odds Ratio (OR) 1.73, 95% Confidence Interval (CI) 1.05-2.85; p = 0.031), pathological T3-4 status compared to the reference category pT0-2 (OR 2.68; 95% CI 2.00-3.59; p < 0.001), PSA level 10–20 and >20 compared to the reference category <10 (OR 1.52, 95% 1.06-2.17; p = 0.023, and OR 1.80, 95% 1.06-3.05; p = 0.029, respectively), Gleason score 7 and 8–10 compared to the reference category 2–6 (OR 1.61, 95% 1.23-2.12; p = 0.001 and OR 2.25, 95% 1.39-3.63; p = 0.001, respectively), and tumour percentage ≥10% compared to the reference category of <10% (OR 2.90; 95% CI 2.21-3.81; p < 0.001).

**Table 2:** **Multiadjusted comparison** ^**a**^ **of sociodemographic, treatment and surgeon characteristics among prostate cancer patients after prostatectomy (n = 1’254) according to surgical margin status**

Characteristics	Surgical margin status				Adjusted	P-value
					Odds ratio	
	Positive		Negative ^b^			
	(cases)		(controls)			
	N	%	N	%	(95% CI)	
**Period**						
1990-1994	29	50.0	29	50.0	1	0.025
1995-1999	105	42.9	140	57.1	1.05 (0.53-2.06)	
2000-2004	201	40.8	292	59.2	1.47 (0.73-2.99)	
2005-2008	144	31.4	314	68.6	0.92 (0.44-1.92)	
**Type of surgery**						
Perineal/Retropubic	196	45.9	231	54.1	1	0.443
Laparoscopic	62	39.2	96	60.8	0.96 (0.61-1.53)	
Robot-assisted	35	32.1	74	67.9	0.96 (0.55-1.69)	
Unknown	186	33.2	374	66.8	0.77 (0.52-1.12)	
**Clinical T**						
cT0-cT1	83	31.8	178	68.2	1	0.038
cT2	163	35.7	293	64.3	0.93 (0.65-1.31)	
cT3	79	63.2	46	36.8	1.73* (1.05-2.85)	
cTx	154	37.4	258	62.6	1.18 (0.82-1.70)	
**Pathological T**						
pT0-pT2	239	27.5	629	72.5	1	<0.001
pT3-pT4	240	62.2	146	37.8	2.68 (2.00-3.59)	
**PSA level**						
0-9	217	32.8	445	67.2	1	0.036
10-20	98	51.0	94	49.0	1.52* (1.06-2.17)	
21+	57	64.0	32	36.0	1.80* (1.06-3.05)	
Unknown	107	34.4	204	65.6	1.03 (0.74-1.42)	
**Gleason**						
2-6	218	30.5	497	69.5	1	<0.001
7	195	45.2	236	54.8	1.61*** (1.23-2.12)	
8-10	60	61.9	37	38.1	2.25*** (1.39-3.63)	
Unknown	6	54.5	5	45.5	1.87 (0.46-7.71)	
**Tumour percentage** ^**c**^						
≤10%	202	26.7	554	73.3	1	<0.001
>10%	252	57.9	183	42.1	2.90*** (2.21-3.81)	
Unknown	25	39.7	38	60.3	1.37 (0.78-2.43)	
**Total**	**479**	**38.2**	**775**	**61.8**		

Geneva Cancer Registry 1990–2008.

^a^Logistic regression adjusted for all variables (clinical and pathological T status were entered separately, due to their co-linearity; the results presented derive from the model with clinical T).

^b^Negative and close.

^c^Tumour percentage of the prostate was calculated as tumour volume divided by the prostate volume.

CI: confidence interval.

*p < 0.05; ***p < 0.001.

### Association between post-operative surgical margins and prostate cancer-specific survival

**Figure 1:**
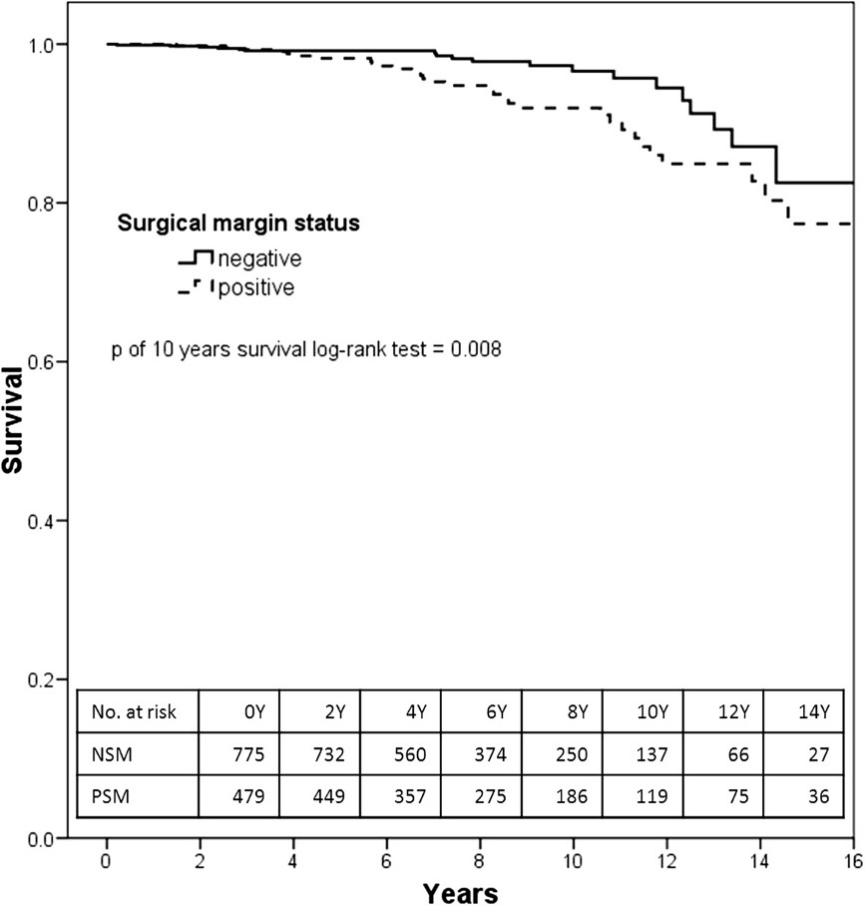
**Kaplan Meier curve of the prostate cancer-specific survival among prostate cancer patients after radical prostatectomy according to surgical margin, Geneva Cancer Registry, 1990–2010.** No. at risk: Numbers of persons at risk at the beginning of the period of follow-up. NSM: Negative Surgical Margin. PSM: Positive Surgical Margin.

During the follow-up period, 141 of the 1,254 men died (11%) including 51 (4%) from prostate cancer. Patients lost to follow-up were 153/1,254 (12.2%). The mean follow-up time was 6.9 years and the median time was 6.1 years (range two months-20 years). Ten-year PCSS was 96.6% (95% CI: 94.3-98.8) for the negative surgical group, and 92.0% (95% CI: 88.5-95.4) for the PSM group (log rank p = 0.008) (Figure 1). Within the negative group the 10-year survival of patients with a truly negative margin was 97.1% (95% CI: 94.6-99.5) and that of patients with close margins was 94.5% (95% CI: 88.8-99.9). In the univariate Cox analysis, surgical margin status, age, surgeon caseload, pathological T-stage, PSA level, Gleason score and tumour percentage were significantly associated with prostate cancer-specific survival (Table 3). In the multivariate Cox regression including the entire set of variables tested in crude analysis and using stepwise backward elimination, age, pathological T-status, PSA level and Gleason score remained significantly associated with PCSS (Table 3). Men in the age category 60–69 (HR 0.28; 95% CI 0.11-0.66; p = 0.004) had a lower risk of mortality compared to the reference category of <60 years. Men with a pathological T3-4 status had a higher risk of mortality compared to the reference category pT0-2 (HR 2.61; 95% CI 1.05-6.48; p = 0.039), as well as men with PSA level greater than 21 (HR 2.89; 95% CI 1.18-7.08; p = 0.020) and a Gleason score 7 (HR 5.28; 95% CI 1.67-16.7; p = 0.005), 8–10 (HR 22.53; 95% CI 7.10-71.5; p < 0.001) and an unknown Gleason score (HR 10.59; 95% CI 1.04-108; p = 0.046) had a higher risk of mortality compared to the reference category 2–6. Yet, surgical margin status was not any longer significantly associated with prostate cancer-specific mortality.

**Table 3:** **Prognostics factors of 10 years prostate cancer mortality derived from Cox regression analysis among prostate cancer patients with prostatectomy**

	Total	PC specific death		Unadjusted hazard ratio			Adjusted hazard ratio ^a^		
	N	N	%	HR	(95% CI)	p-value	HR	(95% CI)	p-value
Surgical Margin									
Negative^b^	775	12	1.6	1		0.010			
Positive	479	21	4.4	2.54*	(1.25-5.16)				
Age class									
<60	342	12	3.5	1		0.004	1		0.001
60-69	721	10	1.4	0.39*	(0.17-0.91)		0.28**	(0.11-0.66)	
≥70	191	11	5.8	1.68	(0.74-3.81)		1.31	(0.56-3.02)	
Period									
1990-99	303	17	5.6	1		0.156			
2000-08	951	16	1.7	0.60	(0.29-1.22)				
Method of detection									
Screening	1046	23	2.2	1.00		0.109			
Symptoms	97	8	8.3	2.34*	(1.04-5.28)				
Other	111	2	1.8	0.89	(0.21-3.76)				
Surgeon caseload									
High (>50)	1007	18	1.8	1		0.006			
Middle (15–50)	166	12	7.2	3.68***	(1.78-7.63)				
Low (<15)	75	3	4.0	2.31	(0.68-7.84)				
Unknown	6	0	0.0	-					
Clinical T									
cT0-cT1	261	2	0.8	1		0.359			
cT2	456	13	2.9	2.92	(0.66-13.0)				
cT3	125	6	4.8	4.33	(0.87-21.5)				
cTx	412	12	2.9	3.06	(0.69-13.7)				
Pathological T									
pT0-pT2	868	7	0.8	1		<0.001	1		0.0387
pT3-pT4	386	26	6.7	6.97***	(3.02-16.1)		2.61*	(1.05-6.48)	
PSA level									
0-9	662	10	1.5	1		<0.001	1		0.0379
10-20	192	7	3.7	1.71	(0.65-4.52)		1.02	(0.38-2.76)	
21+	89	12	13.5	6.40***	(2.75-14.9)		2.89*	(1.18-7.08)	
Unknown	311	4	1.3	0.63	(0.20-2.01)		0.76	(0.23-2.46)	
Gleason									
2-6	715	4	0.6	1		<0.001	1		<0.001
7	431	12	2. 8	6.59**	(2.12-20.5)		5.28**	(1.67-16.7)	
8-10	97	16	16.5	37.8***	(12.6-113)		22.53***	(7.10-71.5)	
Unknown	11	1	9.1	12.6*	(1.40-113)		10.59*	(1.04-107)	
Tumour percentage^c^									
<10%	756	6	0.8	1		<0.001			
≥10%	435	24	5.5	5.88***	(2.40-14.4)				
Unknown	63	3	4.8	4.46*	(1.11-17.9)				
Radiotherapy^d^									
No	1067	23	2.2	1		0.038			
Yes	187	10	5.4	2.19*	(1.04-4.61)				
Total	1254	33	2.63						

Geneva Cancer Registry 1990–2008.

^a^Adjusted HRs were calculated using Cox Backward Regression including the entire set of variables tested in crude analysis, except clinical T.

^b^Negative and close.

^c^Radiotherapy includes both salvage and adjuvant categories.

^d^Tumour percentage of the prostate was calculated as tumour volume divided by the prostate volume.

CI: Confidence interval.

PC: Prostate Cancer.

*p < 0.05; **p < 0.01; ***p < 0.001.

## Discussion

In the current study, we found that more aggressive tumour characteristics are responsible for both PSM after prostatectomy and worse prostate cancer-specific survival. Although, surgical margin status was associated with prostate cancer-specific survival in univariate analysis with PSM patients showing a doubled risk of dying as compared to NSM patients, this increased risk disappeared after adjusting for variables related to the gravity of the disease in the multivariate analysis. Yossepowitch *et al.*, have recently published a systematic review of the literature covering the last ten years, on positive surgical margins after radical prostatectomy evaluating also their oncologic impacts [22]. The authors concluded that the long term impact of positive margins on cancer progression and specific survival is highly variable and largely dependent on additional risk modifiers. In fact, they noted that while all the revised studies showed a significant association between positive margins and biochemical recurrence, the data pertaining to metastatic progression and death were less consistent with only two studies indicating that PSMs were significantly associated with an increased risk of prostate cancer-specific mortality [15, 23]. In the Surveillance, Epidemiology and End Results (SEER) data analysis, Wright *et al*., found a 2.6-fold increased unadjusted risk of prostate cancer-specific mortality that remained significant also after adjusting for grade, stage, additional radiotherapy, age, race, registry and year of diagnosis. However, when stratified by adverse pathological features of the tumour these findings held only for those with higher grade or pT3 tumours [15]. In a more recent study on a single surgeon cohort, Chalfin *et al*., showed that PSM had a statistically significant, but modest, adverse effect on prostate cancer-specific mortality in a model including Gleason score, year of surgery and pathological stage [23]. Similarly to these studies, in our study we found a strong association of prostate cancer-specific mortality with PSM in univariate analysis, however, this association disappeared in the multivariate analysis. Unlike these studies in our multivariate model we simultaneously adjusted for all the pathological adverse tumour features, including Gleason, stage, PSA level, percent of the tumour in the prostate, which emerged as the strongest predictors of mortality. Consistently with the results of another recent study, our results suggest that PSM alone does not increase the risk of dying from prostate cancer [24].

In the literature, surgical margins after prostatectomy are evaluated in different ways through different study designs. Population based studies are valuable designs mostly because of the large cohorts involved, however the accuracy is often not very high. After a systematic audit of the SEER registry, Shah *et al*., showed that 30% of radical prostatectomies performed in 2007 were inaccurately coded, and concluded that clinicians and investigators should recognize the limitations of tumour registry data on PSM [25]. For the current study, we opened all the files and re-checked all parameters and information, improving the accuracy of our compared to the available literature based on cancer registries. This could explain why the overall proportion of 38.2% PSM is higher, compared to the range found in other studies [5]. The proportion of PSM decreased during the study period, from 50% in 1990–1994, to 31% in 2005–2008. Stage shift and surgeons at the end of the learning curve of prostatectomy, may explain the finding of significantly lower PSM rates in the later prostatectomies. In fact, we found that the ratio of cancer with pathological T0-T2/T3-T4 went from 0.7 in 1990–1994, to 3.4 in 2005–2008 (test for trend = 54.58, p < 0.001). On the other hand, we also observed in the univariate analysis that "closed" surgeries such as laparoscopic and robot-assisted surgeries yielded significantly less PSM, confirming the findings of Touijer *et al*. [26] However, for the cases treated in the private sector, the surgical procedure was not always reported. Also, most robot-assisted surgeries reported in our study were performed in the public hospital by a selected group of surgeons. The number of deaths from prostate cancer in the groups of surgery procedures was too low to allow showing an association with survival.

The present study was a retrospective analysis with a relative short follow-up, which is the main limitation of the study. This could be the main reason for the absence of impact of PSM on PCSS. If we accept that BCR is a surrogate of PCSS, since the influence of PSM on BCR is well established, we could foresee that only a study with a median time of follow-up > 10 years would be able to highlight PSM as a significant predictor of PCSS on a multivariate basis. However, we could not provide data regarding biochemical recurrences, as the Geneva Cancer Registry does not collect this information. Furthermore, the number of prostate cancer deaths was very low (n = 33 at 10 year and n = 51 at the end of follow-up), another possible reason for underpowered results. However, we observed similar survival rates in other studies [14, 15]. Another limitation is the use of different pathology laboratories (private and public) in the area. This could have caused inter-observer variability, which was evident in the classification of close surgical margins: 72% were reported from the private pathology laboratory and 28% from the public laboratory. Because of the limited number of patients with close surgical margins, those patients were regrouped together with the negative surgical margins in further analyses, as already suggested in the literature [21]. Another limitation of the study is the relatively high percentage of patients lost to follow-up (12.2%). This is peculiar to the Geneva canton, related to the fact that Geneva is an international city, with 43% of the population consisting of foreigners and a strong work-related migration rate. We compared the patients lost to follow-up to the rest of the study population and found no significant differences in terms of patient, tumour and treatment characteristics. Therefore, we find it unlikely that this selection could have strongly biased our results in terms of the impact of PSM on mortality. On the other hand, because 12% of the cohort was not at risk of prostate cancer mortality, the substantial number of patients who were lost to follow-up could be one of the reasons for the high specific survival rate that we observed. As we had only partial information on the presence of comorbidities, this variable was not included in our analysis. However, in this population comorbidities are expected to have had only a limited impact on survival as the population is younger and therefore healthier; among the 231 study patients for whom information on comorbidities was available, only 32% reported at least one comorbidity, as opposed to 51% of all prostate cancer patients aged >65 years in our registry. Another variable we did not include in the analyses, as available only since 2008, was the information about nerve-sparing approach, however, previous studies have shown that nerve-sparing procedures are safe and not associated with an increased positive margin rate nor on disease progression [27]. The strength of our study is that the population-based approach permits both to generalize the results to the whole population and to examine the effect of PSM in routine care. In addition, the re-opening of all the patients’ files gave further strength to this study, since we were able to double-check and validate all the data. If we consider the context of data collection in a cancer registry, one could generally recommend, as recently stated by Raldow *et al*., that the extension of the treatment registration period from six to nine months or even one year, especially in the case of prostate cancer treatment, could be an advantage for future studies like this one [28]. Finally, in our analyses we could take into account of a large number of important prognostic factors for prostate cancer mortality.

Although the association between PSM and survival is not clear, the link with biochemical recurrence is well established [9–12]. Indeed, in the 2011 European Association of Urology guidelines, as well as the National Comprehensive Cancer Network (NCCN) guidelines, it has been recommended to take into account surgical margin status in the adjuvant treatment decision, as an independent risk factor for BCR [29, 30]. In a recent publication, Lu *et al*., found that close surgical margins as well were independently associated with a twofold risk of postoperative biochemical recurrence and suggest that close margins might be an indicator of local recurrence, and therefore also be of relevance when considering salvage therapy [31]. To date only the SWOG 8794 trial with its long median follow-up could demonstrate improved overall survival with adjuvant radiation therapy in men with pT3a and/or positive margin disease in exploratory analysis. However, the differential effect of adjuvant radiotherapy vs early salvage radiotherapy was not evaluated in this trial, as salvage at PSA recurrence was not mandated by protocol in the observation arm ( [32]). In a situation where the scientific evidence in favour of early *vs.* delayed use of radiotherapy to control local recurrence is still lacking, it seems reasonable to add the information on PSM to the other clinical prognostic variables in order to obtain a more comprehensive assessment of the individual risk [22].

## Conclusions

To conclude, our data showed that surgical margins after prostatectomy are predominantly predicted by tumour characteristics, but are not independently associated with prostate cancer-specific survival. Aggressive tumour characteristics are correlated with both positive surgical margins after prostatectomy and worse prostate cancer-specific survival. These results should be considered for future research and when evaluating patients with a PSM for adjuvant and/or salvage therapy.

## Abbreviations

PSM: Positive surgical margin
NSM: Negative surgical marigin
PCSS: Prostate cancer-specific survival
PSA: prostate specific antigen screening
BCR: Breast cancer recurrence
TNM: Tumour node metastasis
SD: Standard deviation
OR: Odds ratio
CI: Confidence interval
HR: Hazard ratio
SEER: Surveillance, Epidemiology and End Results.
